# TNF-dependent regulation and activation of innate immune cells are essential for host protection against cerebral tuberculosis

**DOI:** 10.1186/s12974-015-0345-1

**Published:** 2015-06-26

**Authors:** Ngiambudulu M. Francisco, Nai-Jen Hsu, Roanne Keeton, Philippa Randall, Boipelo Sebesho, Nasiema Allie, Dhirendra Govender, Valerie Quesniaux, Bernhard Ryffel, Lauriston Kellaway, Muazzam Jacobs

**Affiliations:** Division of Immunology, Department of Clinical Laboratory Science, Institute of Infectious Disease and Molecular Medicine, Faculty of Health Sciences, University of Cape Town, Observatory, Cape Town, 7925 South Africa; Division for Postgraduate Studies, University of the Western Cape, Bellville, South Africa; Division of Anatomical Pathology, Faculty of Health Sciences, University of Cape Town, Cape Town, South Africa; National Health Laboratory Service, Johannesburg, South Africa; Experimental and Molecular Immunology and Neurogenetics, University of Orleans, Orleans, France; CNRS UMR7355, Orleans, France; Department of Human Biology, Faculty of Health Sciences, University of Cape Town, Cape Town, South Africa

**Keywords:** *Mycobacterium tuberculosis*, Infection, Tumour necrosis factor, Neuron

## Abstract

**Background:**

Tuberculosis (TB) affects one third of the global population, and TB of the central nervous system (CNS-TB) is the most severe form of tuberculosis which often associates with high mortality. The pro-inflammatory cytokine tumour necrosis factor (TNF) plays a critical role in the initial and long-term host immune protection against *Mycobacterium tuberculosis* (*M. tuberculosis*) which involves the activation of innate immune cells and structure maintenance of granulomas. However, the contribution of TNF, in particular neuron-derived TNF, in the control of cerebral *M. tuberculosis* infection and its protective immune responses in the CNS were not clear.

**Methods:**

We generated neuron-specific TNF-deficient (NsTNF^−/−^) mice and compared outcomes of disease against TNF^f/f^ control and global TNF^−/−^ mice. Mycobacterial burden in brains, lungs and spleens were compared, and cerebral pathology and cellular contributions analysed by microscopy and flow cytometry after *M. tuberculosis* infection. Activation of innate immune cells was measured by flow cytometry and cell function assessed by cytokine and chemokine quantification using enzyme-linked immunosorbent assay (ELISA).

**Results:**

Intracerebral *M. tuberculosis* infection of TNF^−/−^ mice rendered animals highly susceptible, accompanied by uncontrolled bacilli replication and eventual mortality. In contrast, NsTNF^−/−^ mice were resistant to infection and presented with a phenotype similar to that in TNF^f/f^ control mice. Impaired immunity in TNF^−/−^ mice was associated with altered cytokine and chemokine synthesis in the brain and characterised by a reduced number of activated innate immune cells. Brain pathology reflected enhanced inflammation dominated by neutrophil influx.

**Conclusion:**

Our data show that neuron-derived TNF has a limited role in immune responses, but overall TNF production is necessary for protective immunity against CNS-TB.

## Background

Tuberculosis of the central nervous system (CNS-TB) is associated with significant mortality and high distressing levels of neurological morbidity, the majority of survivors suffering permanent neurologic complications [1–3]. It disproportionately affects children, especially in the developing world and immunocompromised individuals despite the availability of anti-tuberculosis treatment [3–5]. The lengthy duration of therapy for CNS-TB makes adherence difficult. In Sub-Saharan African and some Asian countries, tuberculosis is now one of the most common forms of bacterial meningitis due to the effects of HIV [6–8].

CNS-TB results from a rupture of subpial or subependymal foci which has been deposited earlier following lympho-haematogenous dissemination of *Mycobacterium tuberculosis* from the primary pulmonary infection, or rupture of an adjacent parenchymal focus [1, 9]. It is widely accepted that bacilli reach the CNS by a haematogenous route secondary to disease elsewhere in the body. Since the brain parenchyma and the meninges are physiologically and anatomically protected from the systemic circulation system by the blood–brain barrier (BBB), the mechanisms by which the bacilli initially invade this barrier are not clear. It is believed that *M. tuberculosis* can cross the BBB as a free organism or via infected peripheral myeloid cells [10]. However, the latter hypothesis seems controversial, as myeloid cellular traffic is severely restricted into the CNS prior to invasion by *M. tuberculosis* [11], however lymphoid cells in physiological state can bypass the BBB by entering the subarachnoid space via the meningeal veins or the choroid plexus [12, 13].

Tumour necrosis factor (TNF) has been shown to be critical in the pathogenesis of *M. tuberculosis* in the central nervous system [14–16]. TNF is synthesised by several cell types of haematopoietic origin, such as microglia/macrophages, neutrophils, dendritic cells and lymphocytes, and non-haematopoietic origin such as astrocytes and neurons [17–20]. The production of TNF in the CNS alters the BBB permeability and CSF leukocytosis in experimental bacterial meningitis [21, 22] and has been implicated in fostering the progression of CNS-TB in a rabbit model [16]. However, Tobin et al. [15] have shown using human and zebrafish models that either state, TNF deficiency or excessive TNF production, causes macrophage lysis, therefore placing *M. tuberculosis* in a permissive extracellular niche [15, 23, 24]. Within this context, it is important to understand the role of TNF in host immunity during *M. tuberculosis* infection in the CNS. Here, we generated neuron-specific TNF-deficient (NsTNF^−/−^) mice and investigated outcomes after intracerebral *M. tuberculosis* infection in comparative studies with TNF^f/f^ control and global TNF^−/−^ mice.

## Methods

### Mice

All mouse strains, TNF^f/f^ (TNF-floxed wild-type [25]), Syn1-Cre (synapsin I promoter-driven Cre-transgenic, obtained from the Jackson Laboratory, http://jaxmice.jax.org/strain/003966.html), NsTNF^−/−^ (neuron-derived TNF-deficient) and TNF^−/−^ (TNF-deficient [25]) mice, were bred and maintained under specific pathogen-free conditions at the animal facility of the University of Cape Town (South Africa). Adult mice between 8 and 12 weeks of age were used, and infected mice were maintained under biosafety level 3 conditions. All animal procedures were approved by the Animal Research Ethics Committee (AEC reference number: 010/018), University of Cape Town, in accordance with the South African National Standard.

### Genotyping PCR analysis

Genotyping of mouse strains was performed by PCR analysis of DNA extracted from tail biopsies. Genetic analysis of TNF^f/f^ mice was previously described [25]. Syn1-Cre and NsTNF^−/−^ mouse were genotyped for Cre expression using the primer sets IMR1084 (5′ GCG GTC TGG CAG TAA AAA CTA TC 3′) and IMR1085 (5′ GTG AAA CAG CAT TGC TGT CAC TT 3′), and the internal controls IMR7338 (5′ CTA GGC CAC AGA ATT GAA AGA TCT 3′) and IMR7339 (5′ GTA GGT GGA AAT TCT AGC ATC ATC C 3′). For the presence of the TNF loxP gene in NsTNF^−/−^ mice, primers KO41 (5′ TGA GTC TGT CTT AAC TAA CC 3′) and KO42 (5′ CCC TTC ATT CTC AAG GCA CA 3′) were used [25].

### Aerosol infection and immunohistochemistry of brain tissue

*M. tuberculosis* with green fluorescent expressing protein (*M. tuberculosis* H37Rv-GFP, provided by Joel Ernst, New York University School of Medicine, USA) was grown in Middlebrook 7H9 medium (Difco™ Laboratories) containing 25 μg/ml kanamycin and 0.5 % glycerol and enriched with 10 % OADC. Bacterial cultures were incubated at 37 °C and grown until log phase, aliquoted and stored at −80 °C. A frozen aliquot of *M. tuberculosis* was thawed, passed 30 times through a 29-G needle and diluted in sterile saline. Mice were infected by aerosol inhalation at a dose of 200–500 colony-forming units (CFUs)/lung under biosafety level 3 conditions using a Glas-Col Inhalation Exposure System Model A4224. For analysis, infected mice were deeply anaesthetised and transcardially perfused with 4 % paraformaldehyde. Brains were sectioned at 40 μm using a vibratome and processed for immunohistochemical staining. Neurons were labelled with polyclonal anti-MAP2 (1 μg/ml, Abcam) or anti-β-III-tubulin antibody (1 μg/ml, Abcam), and anti-rabbit Cy3-conjugated secondary antibody (1.5 μg/ml, Jackson ImmunoResearch Laboratories). The microglia/monocytes were labelled with monoclonal anti-CD11b antibody (Clone M1/70, 1 μg/ml, Abcam) and anti-rat Cy3-conjugated secondary antibodies (1.5 μg/ml, Jackson ImmunoResearch Laboratories). The sections were incubated with nuclear marker DAPI (Sigma) and then mounted in fluorescent mounting medium and images captured using a Zeiss 510LSM unit.

### Intracerebral infection and clinical scoring

*M. tuberculosis* strain H37Rv was grown at 37 °C in Middlebrook 7H9 broth containing 10 % OADC and 0.5 % Tween 80 until log phase, then aliquoted and stored at −80 °C. A frozen aliquot was thawed, passed 30 times through a 29-G needle and diluted in sterile saline. Intracerebral infection was performed using a stereotaxic approach of directly injecting *M. tuberculosis* H37Rv into the cerebral cortex. Prior to inoculation, a small burr hole was constructed anterior to the bregma and to the left of the midline in the skull exposing the dura mater. Five mice per strain were inoculated intracerebrally with 1 × 10^3^–1 × 10^4^ CFUs of *M. tuberculosis* H37Rv using a Hamilton syringe (Gastight no. 1701, Switzerland). The burr hole was sealed with bone wax and the skin sutured. Animals received a prophylactic pain killer for 3 days at 24-h intervals. The clinical scoring system was adapted from previous reports [26, 27]. Here, mice were weighed and scored daily for neurologic manifestations during the course of infection as follows: normal (no detectable signs) = 0; head tilt = 1; motility or decrease activity = 2; behaviour depression = 3; and moribund state = 4. Organs of infected mice were harvested and processed at 1, 2, 3 and 15 weeks post-infection. Haematoxylin and eosin (H&E)-stained brain sections at 3 weeks post-infection were reviewed and analysed by a pathologist who was blinded to the study.

### Colony enumeration assay

Bacterial burdens in the brains, lungs and spleens of infected mice were determined at specific time points after infection with *M. tuberculosis*. Organs were weighed and homogenised in 0.04 % Tween 80 saline. Tenfold serial dilutions of organ homogenates were plated in duplicates on Middlebrook 7H10 (Becton, Dickinson and Company) agar plates containing 10 % OADC (Life Technologies, Gaithersburg, MD) and were incubated at 37 °C for 19–21 days. The concentration of *M. tuberculosis* was then determined by counting the CFUs.

### Flow cytometry

To determine the expression level of TNF by neurons, TNF^f/f^, NsTNF^−/−^ and TNF^−/−^ mice were intracerebrally stimulated with 5 μg/ml of lipopolysaccharide (LPS) for 90 min. Mice were transcardially perfused with 4 % paraformaldehyde. For single-cell suspensions, brains were isolated and tissue passed through a 70-μm nylon cell strainer (Beckton and Dickinson) and washed 2× with phosphate-buffered saline (PBS) and the cell concentration was determined by counting in the presence of trypan blue. TNF expression in neurons was measured by intracellular staining through the addition of saponin buffer to permeabilise the cells, which were then labelled with polyclonal anti-β-III-tubulin antibody (20 μg/ml, Abcam) and an anti-rabbit PE-conjugated secondary antibody (1.5 μg/ml, Jackson ImmunoResearch Laboratories), and anti-TNF antibody (TNF:Alexa 647, Clone MP6-XT22, BD Pharmingen™).

To analyse surface marker expression in microglia, macrophages and dendritic cells, the following antibodies were used: CD11b:PerCP-Cy5-5 (Clone M1/70, BD Pharmingen™ [2 μg/ml]); CD11c:Alexa 700 (Clone HL3, BD Pharmingen™ [2.5 μg/ml]); CD45:APC (Clone 30-F11, BD Pharmingen™ [2 μg/ml]); CD80:FITC (16-10A1, BD Pharmingen™ [4 μg/ml]); CD86:V450 (Clone GL1, BD Horizon™ [4 μg/ml]) and MHCII/(I-A/I-E):PE (M5/114.15.2, BD Pharmingen™ [2 μg/ml]). Cells were washed with PBS/0.1 % bovine serum albumin (BSA)/0.01 % NaN_3_ and incubated with the appropriate antibodies for 20 min in the dark. Excess antibodies were removed by washing cells 2× with PBS/0.1 % BSA/0.01 % NaN_3_. Pelleted cells were fixed for 18–24 h in 2 % phosphate buffered formaldehyde and analysed on a FACSCalibur (Beckton Dickinson) flow cytometer using CellQuest software. Microglia were defined as CD11b^+^CD45^low^, macrophages as CD11b^+^CD45^high^ and dendritic cells as CD11c^+^CD45^high^ as previously described [28, 29].

### Quantification of chemokines and cytokines

Supernatants from brain homogenates were prepared for cytokine and chemokine measurement by enzyme-linked immunosorbent assay (ELISA) after 3 weeks subsequent to intracerebral *M. tuberculosis* infection. The chemokines MCP-1, MIP-1α and RANTES, and the cytokines IL-1β, IL-12p70 and TNF (R&D Systems, Germany) were measured using commercially available ELISA reagents according to the manufacturer’s instructions. Chemokine and cytokine concentrations were measured by absorbance using a Versamax Microplate Reader (Molecular Devices, LLC, CA) with SoftMax software.

### Statistical analysis

The data are presented as the mean ± standard error of the mean (SD). Statistical analysis was performed by one-way ANOVA and one-tailed *t* test for comparisons amongst the time points. For all tests, a *p* value of ≤0.05 was considered significant.

## Results

### TNF regulates *M. tuberculosis* dissemination into the brain but infection of neurons is TNF independent

**Fig. 1:**
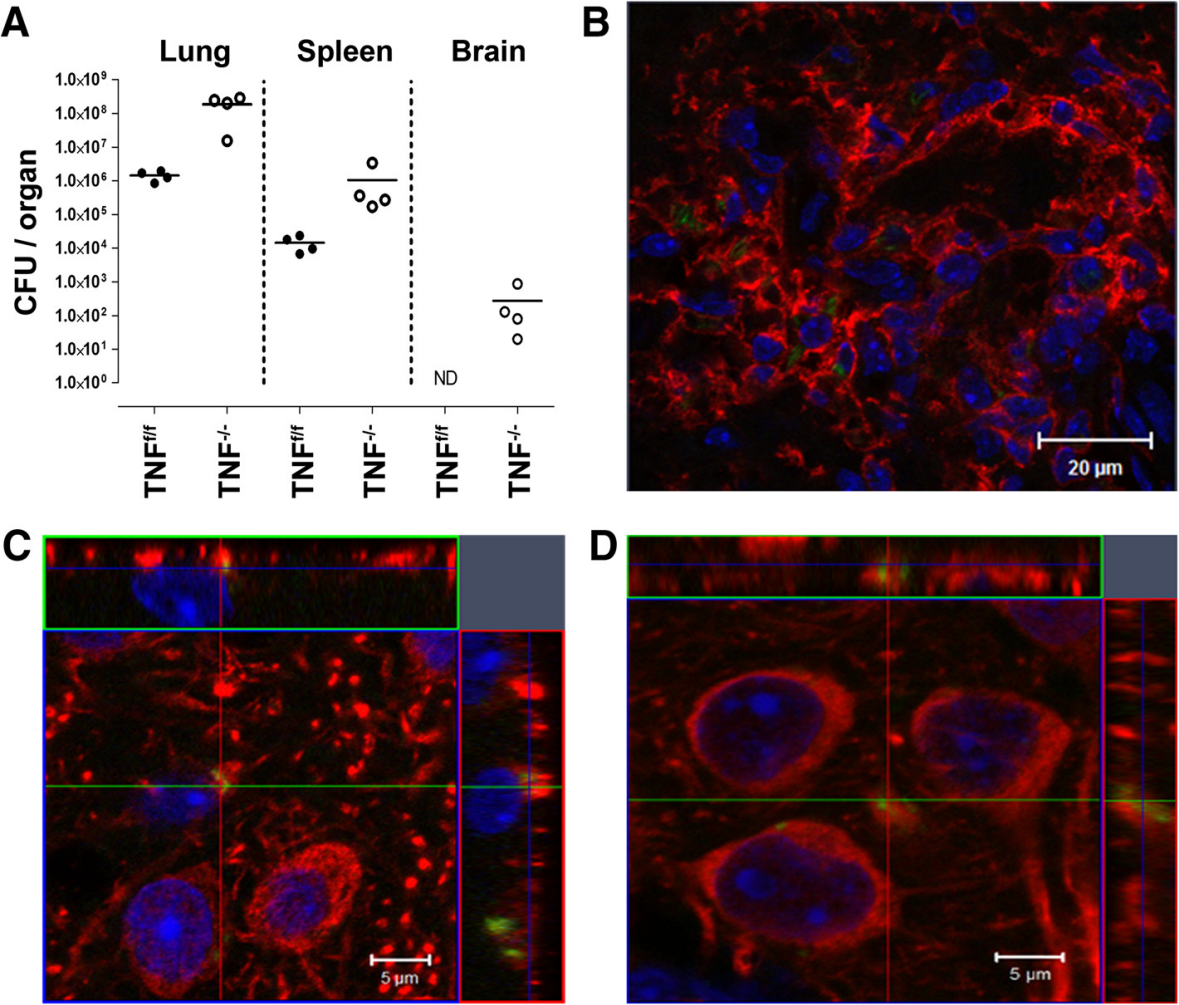
Dissemination of *M. tuberculosis* to the brain in TNF^−/−^ mice. **a** TNF^f/f^ and TNF^−/−^ mice were infected by aerosol inhalation at a dose of 200–500 CFUs/lung of *M. tuberculosis*, and mycobacterial burden in brains, lungs and spleens was determined at day 33 post-infection. **b** Foci of disseminated fluorescent bacilli (*green*) surrounded by CD11b^+^ microglia/macrophages (*red*) were present in the TNF^−/−^ brain parenchyma. **c**, **d** The fluorescent image of intracellular *M. tuberculosis* GFP-expressing bacilli (*green*) found in the neurons (*red*) of TNF^−/−^ mouse, labelled with **c** MAP2 and **d** β-III-tubulin. The orthogonal projection of confocal Z-stacks confirmed the cytosolic location of the bacilli in the *x*–*y* plane. All nuclei were labelled with DAPI (*blue* in **b**–**d**). *Scale bars*: **b** 20 μm; **c**, **d** 5 μm. The data represents two independent experiments

TNF plays an important role in host immunity against *M. tuberculosis* dissemination and latent infection [30–32]. A key function of TNF in granuloma formation and maintenance has been described in models of TNF-gene deficiency or neutralisation [16, 33, 34], supported by clinical evidence observed in the rheumatoid arthritis patients who presented with TB reactivation after anti-TNF therapy [5]. Moreover, inhibition of TNF leads to *M. tuberculosis* dissemination which causes extrapulmonary TB including severe CNS-TB [35, 36]. To investigate whether TNF protects against *M. tuberculosis* dissemination to the CNS, we challenged wild-type (TNF^f/f^) and TNF^−/−^ mice by aerosol inhalation with *M. tuberculosis* H37Rv-GFP. In contrast to TNF^f/f^ mice, TNF^−/−^ mice were unable to control pulmonary TB infection, which resulted in high bacilli burdens in the lungs and spleens (Fig. 1a), similar to previous reports [37, 38]. Further, we confirmed dissemination of *M. tuberculosis* bacilli from the lungs to the brains in the absence of TNF; however, bacilli in the brains of TNF^f/f^ mice were not detectable by culture (Fig. 1a). The latter observation is supported by previous studies of Hernandez Pando et al. [39], who showed limited, if any, detectable bacilli in the brains of wild-type mice using the same *M. tuberculosis* H37Rv laboratory strain.

Pathogenesis of CNS-TB is thought to involve haematogenous spread and phagocytosis of bacilli by microglia which generates a robust immune response [1, 40]. TNF, as a pro-inflammatory cytokine mainly produced by microglia and astrocytes, has been proposed as the main cause of inflammation and neuropathology [2, 40], while others have suggested a neuroprotective role [41]. We have previously demonstrated that neurons can serve as host cells for *M. tuberculosis* [42]. Therefore, to determine if neuron infection is TNF dependent, we investigated whether dissemination of *M. tuberculosis* to the CNS of TNF^−/−^ mice resulted in bacilli infection of neurons. Infected brain tissue sections presented predominantly with bacilli foci enriched with CD11b^+^ microglia/macrophages (Fig. 1b), most of which were intracellular. Significantly, we observed *M. tuberculosis* association with neurons. Three-dimensional orthogonal views of confocal images revealed that the green-expressing bacilli were embedded in the MAP2^+^ (Fig. 1c) or β-tubulin^+^ (Fig. 1d) neuronal cytoplasmic structures, similar to previously described observations in intracranially infected C57Bl/6 mice [42]. The data presented here together with previously published data [37] demonstrates an important role for TNF to regulate pulmonary immune responses against *M. tuberculosis* infection. Failure to control *M. tuberculosis* replication results in bacilli dissemination and subsequent infection of peripheral organs including the central nervous system where infection of neurons can occur independent of TNF.

### Generation and characterisation of NsTNF^−/−^ mice

**Fig. 2:**
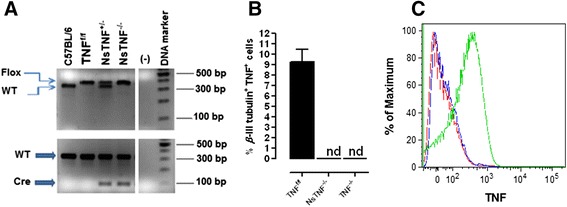
Analysis of NsTNF^−/−^ mice. **a** Deletion of the neuron-specific TNF gene in NsTNF^−/−^ mice was confirmed by PCR analysis. **b** TNF expression in neurons from TNF^f/f^, NsTNF^−/−^ and TNF^−/−^ mice was analysed by flow cytometry after LPS stimulation. **c** Mean fluorescence intensities of TNF expression in TNF^f/f^ (*green*), NsTNF^−/−^ (*blue*) and TNF^−/−^ mice (*red*) are presented

Although our observations suggested TNF-independent neuronal invasion in the CNS, it was reported that TNF production, specifically by neurons, has an impact on disease outcome [43]. To investigate the role of neuron-derived TNF in CNS-TB immunity, we generated a mouse model of neuron-specific TNF deficiency (NsTNF^−/−^) through mating of Syn1-Cre mice [44] and TNF-floxed mice (TNF^f/f^) [25, 37]. We confirmed correct genotypes of the heterozygous Syn1-Cre^Cre/wt^TNF^f/wt^ F1 offspring by PCR analysis. Typically, Syn1-Cre^Cre/wt^TNF^f/wt^ mice displayed the 100-bp amplification product representative of the Cre gene (bottom panel, Fig. 2a) while both the TNF-floxed and normal TNF genes were present, represented by 400- and 350-bp amplification products, respectively (top panel, Fig. 2a). Syn1-Cre^Cre/wt^TNF^f/wt^ F1 offspring was subsequently bred with TNF^f/f^ mice to generate the homozygote neuronal cell type-specific TNF mice (Syn1-Cre^Cre/wt^ TNF^f/f^), and offspring were analysed by tail biopsy analysis. The F2-generation Syn1-Cre^Cre/wt^ TNF^f/f^ mice displayed the 400-bp amplification fragment representative of the TNF-floxed gene while the normal wild-type TNF gene was absent (top panel, Fig. 2a), and the presence of a 100-bp amplification product specified the presence of the Cre gene. The data therefore confirmed the genotype of the new strain which was termed NsTNF^−/−^ mice (bottom panel, Fig. 2a).

We next assessed neuron-specific ablation of TNF. TNF^f/f^, TNF^−/−^ and NsTNF^−/−^ mice were inoculated with 3 μl LPS (5 μg/ml) in the left brain hemisphere by intracerebral inoculation. Single-cell suspensions, generated from harvested brains, were labelled and analysed for neurons expressing TNF. We confirmed expression of TNF in neurons of TNF^f/f^ mice and the absence of TNF in both TNF^−/−^ and NsTNF^−/−^ mice through comparative TNF mean fluorescence intensity analysis which were both negligible in TNF^−/−^ and NsTNF^−/−^ mice (Fig. 2b), indicating almost complete neuron-specific ablation while the MFI for TNF was approximately 100-fold higher in TNF^f/f^ mice (Fig. 2c). Thus, the successful generation of novel NsTNF^−/−^ mice which presented with neuron-specific TNF ablation was confirmed by genetic and flow cytometric analysis.

### TNF^−/−^ mice are highly susceptible to cerebral *M. tuberculosis* infection while NsTNF^−/−^ mice are resistant

**Fig. 3:**
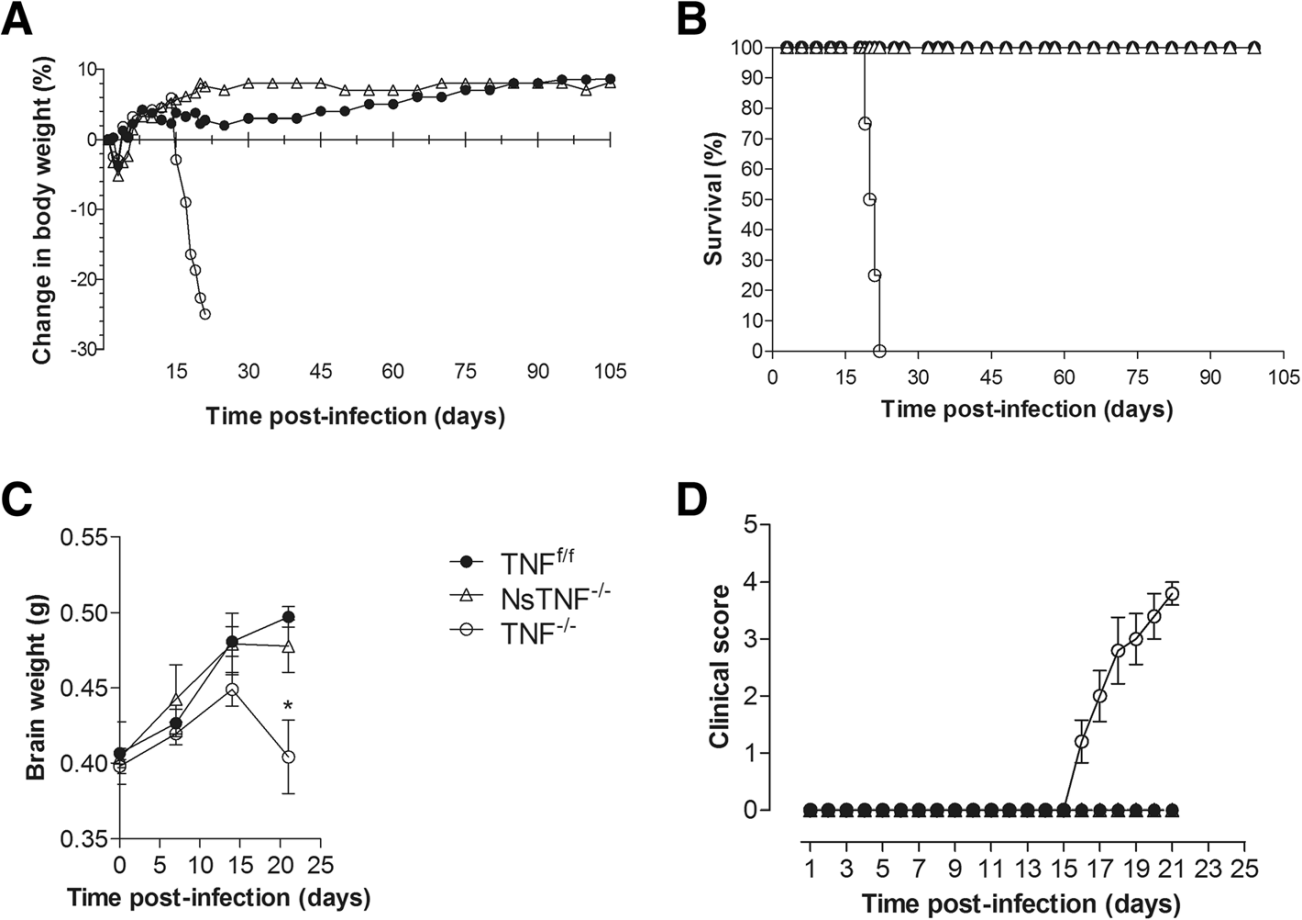
TNF^−/−^ mice are highly susceptible to CNS-TB but NsTNF^−/−^ mice are resistant. TNF^f/f^ (*black circle*), NsTNF^−/−^ (*triangle*), and TNF^−/−^ (*clear circle*) mice were infected with *M. tuberculosis* H37Rv at a dose of 1 × 10^3^–1 × 10^4^ CFUs/brain by intracerebral inoculation. Body weight changes (**a**) and mortality (**b**) were measured and recorded for the experimental duration (*n* = 10–12 mice/group). **c** Brain weights of infected mice were recorded at 1, 2 and 3 weeks post-infection (*n* = 5–6 mice/group). **d** The clinical scores, indicative of the disease severity, are represented (*n* = 10–12 mice/group). The data represents three to five similar experiments. *p ≤ 0.05

Contention exists on the role of TNF in host protection against *M. tuberculosis* infection of the brain. Current literature argues that *M. tuberculosis* persistence is encouraged in the presence of TNF [45] whereas, in pulmonary infection, it is required to control bacterial replication [34, 38, 46]. Also, TNF was shown to be critical for protection against other central nervous system diseases [17, 47]. To resolve the apparent inconsistency in the role of TNF, particularly to address whether TNF contributes to immune protection against *M. tuberculosis* of the central nervous system, and to evaluate neurons as a potential cellular source of TNF in the cerebral immune response, TNF^f/f^, NsTNF^−/−^ and TNF^−/−^ mice were challenged with *M. tuberculosis*. Disease progression was clinically scored, and mortality and changes in body weight were recorded as indications of disease severity (Fig. 3). In contrast to TNF^f/f^ mice which survived infection for 15 weeks, TNF^−/−^ mice were highly susceptible and rapidly succumbed to infection within 3 weeks (Fig. 3b). Initial post-surgery body weight loss and recovery were observed between day 1 and day 5 after *M. tuberculosis* infection as previously reported [48, 49]. Susceptibility was accompanied by rapid loss of >20 % of the original body weight during the last week of illness as opposed to TNF^f/f^ mice which displayed a steady increase in body weight during the same observation period (Fig. 3a). Similar to TNF^f/f^ mice, NsTNF^−/−^ mice were resistant and survived the duration of infection without any measured body weight loss. Brain, lung and spleen weights were measured as representative of organ inflammation during disease development. We observed enhanced brain inflammation in all three strains until 2 weeks post-infection, after which inflammation was sustained in TNF^f/f^ and NsTNF^−/−^ mice but significantly decreased (*p* < 0.05) in TNF^−/−^ mice (Fig. 3a). Neurologic symptoms such as head tilt and anorexia were absent in both TNF^f/f^ and NsTNF^−/−^ mouse strains, contrasting the severe clinical neurologic symptoms prominent (*p* < 0.01) within 16–21 days post-infection in TNF^−/−^ mice (Fig. 3d). It was therefore concluded that TNF was indispensable to generate protective immunity against *M. tuberculosis* infection of the central nervous system and unequivocally established the non-redundancy of TNF as a critical mediator of immune protection against CNS-TB. Nonetheless, while TNF was required for protection, neuron-specific TNF was functionally redundant.

### Cerebral bacilli replication and dissemination are increased in TNF^−/−^ mice but not in NsTNF^−/−^ mice

**Fig. 4:**
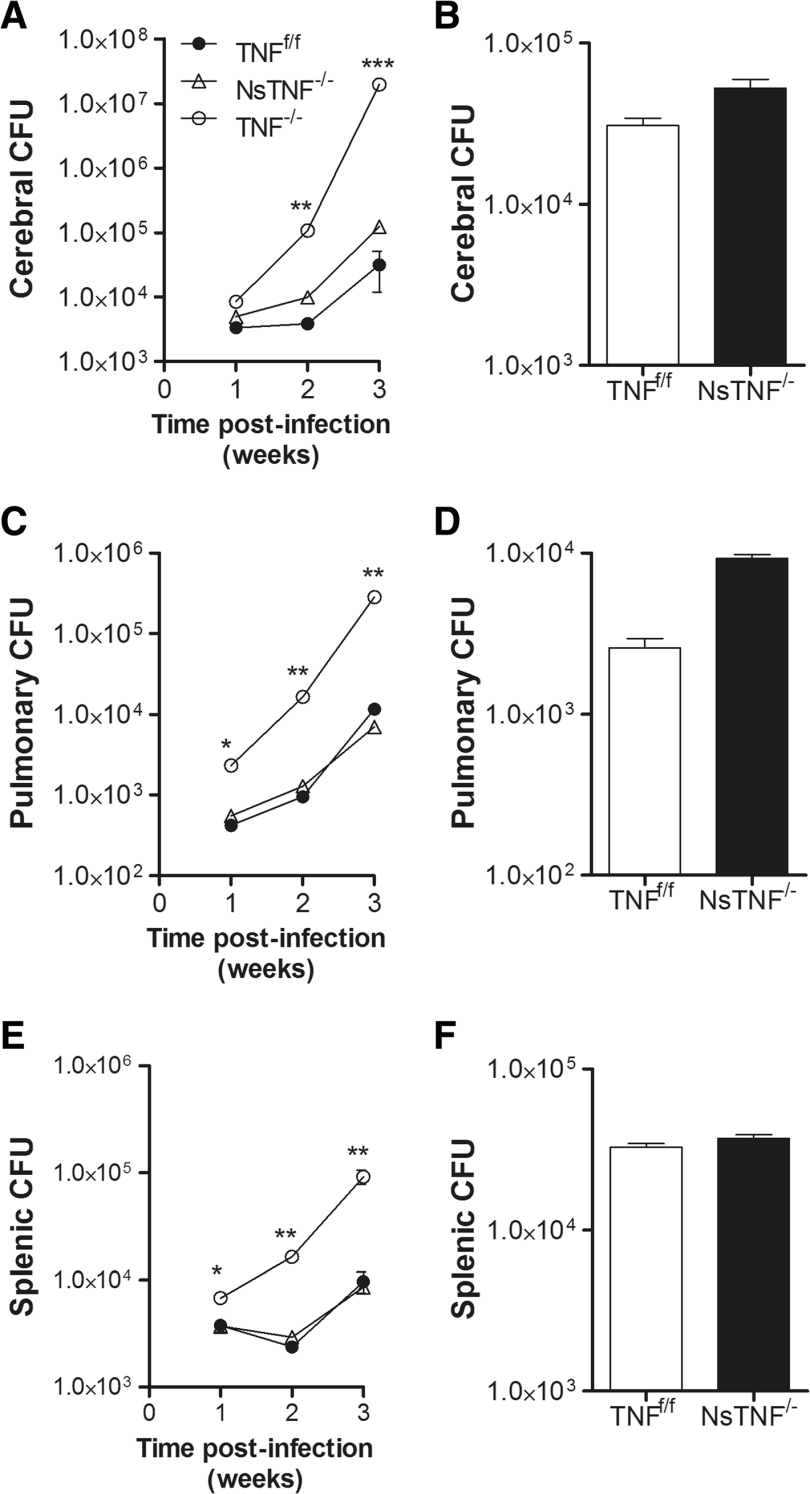
TNF is required to control intracerebral *M. tuberculosis* replication but neuron-derived TNF is redundant. TNF^f/f^ (*black circle*), NsTNF^−/−^ (*triangle*) and TNF^−/−^ (*clear circle*) mice (*n* = 5–6/group) were infected with *M. tuberculosis* at a dose of 1 × 10^3^–1 × 10^4^ CFUs/brain. Bacterial burdens in **a**, **b** brains; **c**, **d** spleens; and **e**, **f** lungs at 1, 2, 3 (*left panel*; **a**, **c**, **e**, respectively) and 15 weeks (*right panel*; **b**, **d**, **e** respectively) post-infection were determined. Data (mean ± SEM of the CFUs of 5–6 mice per time point) are representative of three similar experiments. *p ≤ 0.05; **p ≤ 0.01

**Fig. 5:**
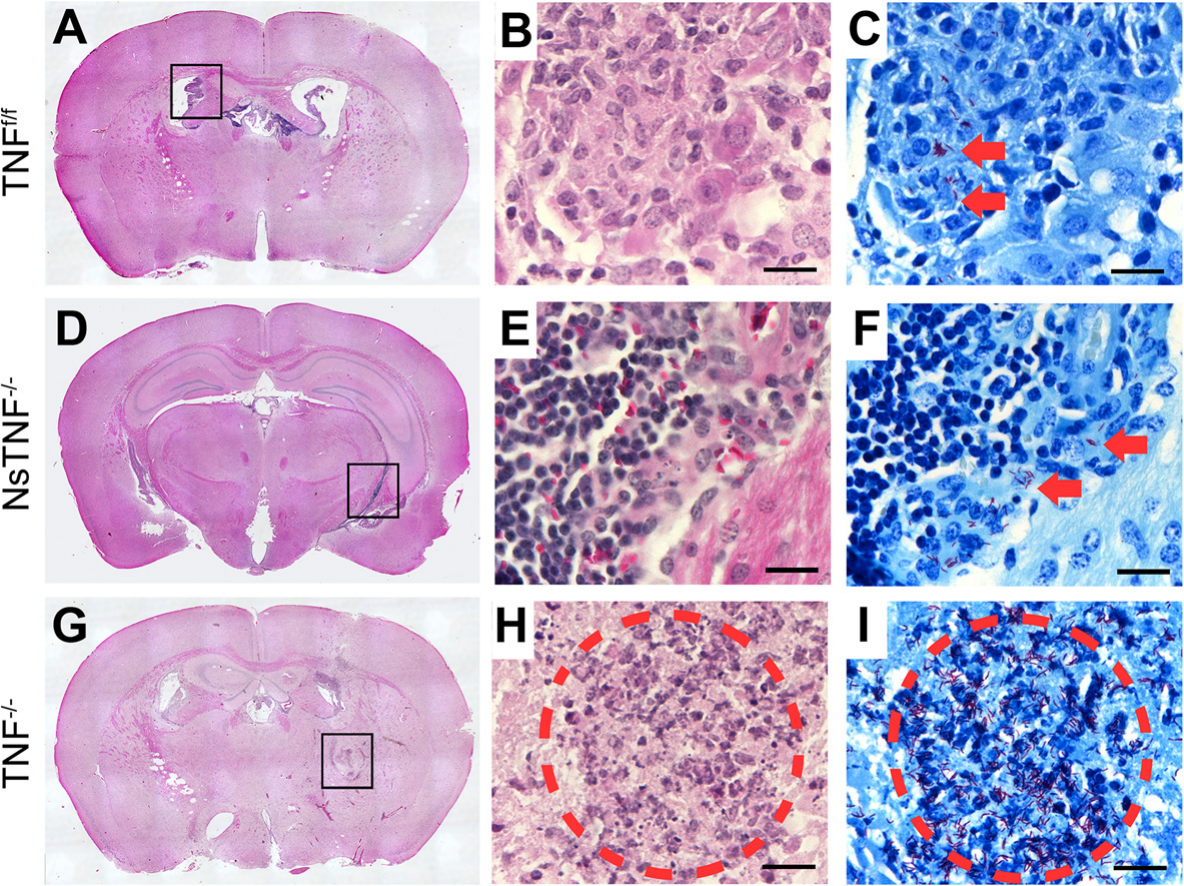
Cerebral pathology is regulated by TNF but neuron-derived TNF is not required. **a**–**c** TNF^f/f^, **d–f** NsTNF^−/−^ and **g**–**i** TNF^−/−^ mice were infected with *M. tuberculosis* H37Rv at a dose of 1 × 10^3^–1 × 10^4^ CFUs/brain by intracerebral inoculation. Haematoxylin and eosin-stained coronal brain tissue sections at 3 weeks post-infection are presented (**a**, **d**, **g**). Ventriculitis of the choroid plexus is primarily lymphocytic in **b** TNF^f/f^ and **e** NsTNF^−/−^ mice while a predominant neutrophil infiltrate accompanied by necrosis is observed in **h** TNF^−/−^ mice. Ziehl–Neelsen staining revealed acid-fast bacilli mainly in the choroid plexus (**c**, *arrow*) and ventricular space (**f**, *arrow*) in TNF^f/f^ and **e** NsTNF^−/−^ mice while infiltration of bacilli was frequently observed in brain parenchyma (**i**) in TNF^−/−^ mice. The granuloma was found in TNF^−/−^ mouse brain (*circle* in **h**, **i**). *Scale bars* 20 μm

The importance of TNF to control cerebral bacilli replication was assessed by comparing pathogen burden in the brains of *M. tuberculosis*-infected TNF^f/f^, NsTNF^−/−^ and TNF^−/−^ mice. We observed an increase in cerebral *M. tuberculosis* replication in TNF^f/f^ mice during acute infection, burdens at week 3 post-infection being significantly higher compared to those at earlier time points (Fig. 4a). In contrast, differences in cerebral bacilli burden were already significantly higher (*p* < 0.01) in TNF^−/−^ mice at 2 weeks post-infection which were sustained at 3 weeks post-infection. In contrast, NsTNF^−/−^ mice controlled acute infection at burden levels equivalent to those in TNF^f/f^ mice and were similarly significantly lower (*p* < 0.01) compared to those in TNF^−/−^ mice (Fig. 4a). We confirmed differences in cerebral bacilli burden by image analysis of acid-fast bacilli staining (Fig. 5). In the brains of TNF^f/f^ and NsTNF^−/−^ mice, acid-fast bacilli were located either in the choroid plexus, in the ventricular space or in the perivascular region at 3 weeks post-infection. However, in TNF^−/−^ mice, necrotic foci containing significantly higher numbers of bacilli were prominent in the brain parenchyma (Fig. 5). We then asked whether neuron-derived TNF is required to control chronic cerebral *M. tuberculosis* infection. We found no difference in cerebral bacilli burdens between TNF^f/f^ and NsTNF^−/−^ mice (Fig. 4b), indicating that TNF synthesis by neurons is not required to control infection. We next measured bacilli burdens in the lungs and spleens of infected mice to evaluate the role of TNF to control *M. tuberculosis* dissemination from the brain. Pulmonary and splenic infection was established early in the absence of TNF and bacilli burdens significantly higher than TNF^f/f^ or NsTNF^−/−^ mice (Fig. 4c, e), indicating that TNF is required to control early dissemination of infection. The comparative equivalence of bacilli burdens in both the lungs and spleens of TNF^f/f^ and NsTNF^−/−^ mice during acute (Fig. 4c, e) or chronic infection (Fig. 4d, f) suggested a redundancy of neuron-derived TNF to contain cerebral spread of infection.

### TNF regulates cerebral inflammation but neuron-derived TNF is not required

We next examined the role of TNF to control cerebral inflammation by analysis of sectioned brain tissue for induced pathology in *M. tuberculosis*-infected mice at 3 weeks post-infection. In the brain sections taken from TNF^f/f^, NsTNF^−/−^ and TNF^−/−^ mice, we observed degrees of inflammation in different regions of the brain in all mouse strains (Fig. 5). Representative images of inflammation in the choroid plexus, ventricles and brain tissue are shown in Fig. 5b, e, h, respectively. Amongst the three mouse strains, TNF^−/−^ mice exhibited the most severe neuropathology and uncontrolled inflammation where acute ventriculitis was characterised by a prominent neutrophil infiltrate which extended into the periventricular tissue (Fig. 5g). The choroid plexus in TNF^−/−^ mice was infiltrated by a mixture of lymphocytes and neutrophils in contrast to a predominantly lymphocytic infiltrate in TNF^f/f^ and NsTNF^−/−^ mice. Also noted were associated foci of necrosis in the adjacent brain tissue with distinct areas of perivascular lymphocyte cuffing (Fig. 5h). Well-formed, discrete granulomas were present in TNF^f/f^ and NsTNF^−/−^ mice unlike in TNF^−/−^ mice where granuloma formation was largely absent.

Therefore, TNF was required to regulate CNS inflammation during *M. tuberculosis* infection; moreover, it determined the quality of the inflammatory response.

### TNF regulates recruitment and proliferation of innate immune cells

**Fig. 6:**
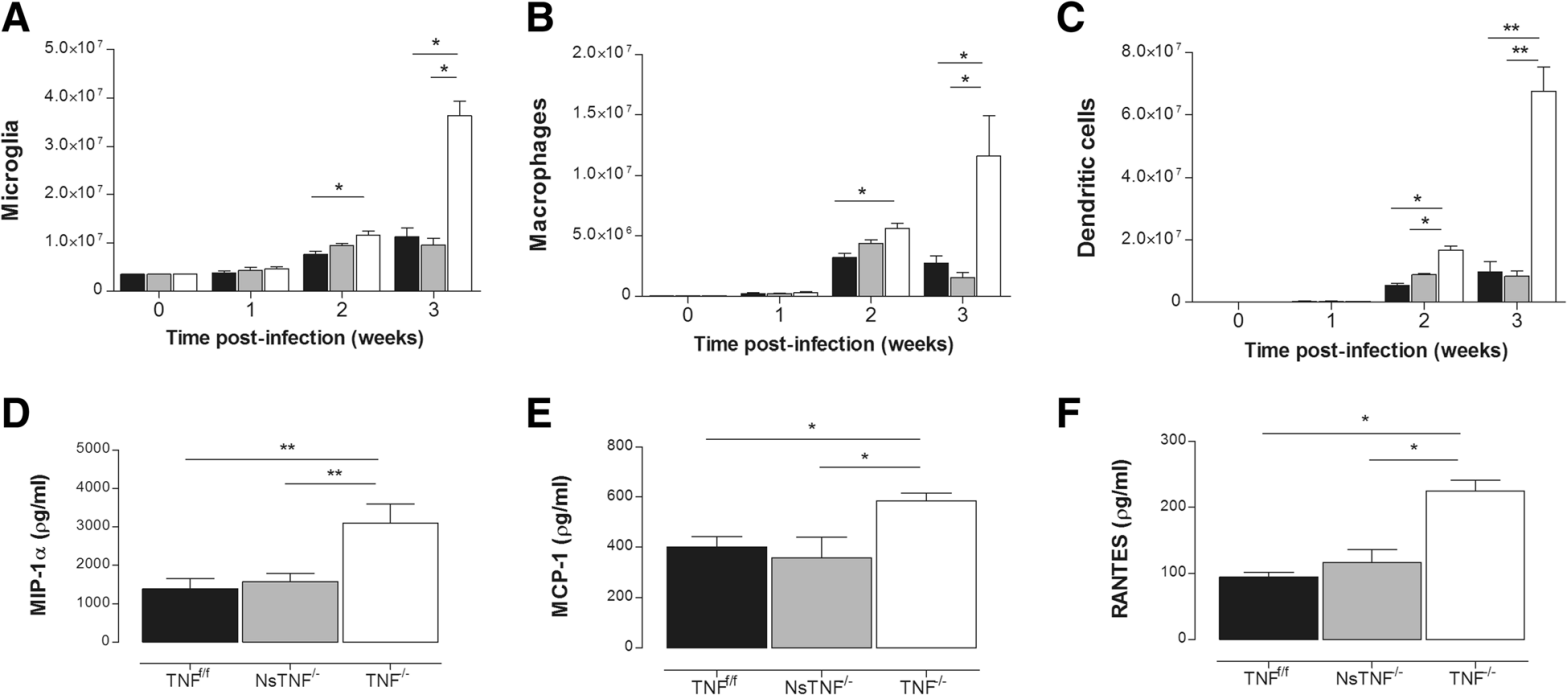
TNF regulates innate immune cell proliferation and recruitment during CNS-TB. TNF^f/f^ (*black*), NsTNF^−/−^ (*grey*) and TNF^−/−^ (*clear*) mice were infected with *M. tuberculosis* H37Rv at a dose of 1 × 10^3^–1 × 10^4^ CFUs/brain by intracerebral inoculation. The number of **a** microglia, **b** macrophages and **c** dendritic cells was analysed by flow cytometry at 0, 1, 2 and 3 weeks in infected brains. The chemokines **d** MIP-1α, **e** MCP-1 and **f** RANTES were measured by ELISA in the brains of infected mice at 3 weeks post-intracerebral infection. Data (mean ± SEM of the CFUs of 5–6 mice per time point) are representative of repeat experiments. *p ≤ 0.05; **p ≤ 0.01

Early susceptibility of TNF^−/−^ mice to cerebral *M. tuberculosis* infection suggested defective TNF-mediated function during initial innate immune responses. We postulated that TNF deficiency may impact on key innate immune cell function to promote the observed susceptible phenotype. We first investigated recruitment of macrophages and dendritic cells to the brain and evaluated proliferation of microglia, as central mediators of innate immune responses subsequent to *M. tuberculosis* challenge. Microglial proliferation and dendritic cell and macrophage recruitment occurred in all three mouse strains in response to *M. tuberculosis* infection (Fig. 6a–c). However, while cellular levels reached maximum at 2 weeks post-infection and were sustained at 3 weeks post-infection in TNF^f/f^ and NsTNF^−/−^ mice, TNF^−/−^ mice displayed significantly higher levels at 2 weeks post-infection, the differential levels enhancing even further at 3 weeks post-infection.

We next investigated whether enhanced innate immune cell recruitment in TNF^−/−^ mice was a consequence of heightened host cerebral chemokine induction in response to *M. tuberculosis* infection. We measured cerebral concentrations of MIP-1α, MCP-1 and RANTES in *M. tuberculosis*-infected mice representative of chemokines known to induce pro-inflammatory effects [50, 51]. We found escalating induction of each chemokine in the brains of infected mice over the duration of the experiment (data not shown). However, while TNF^f/f^ and NsTNF^−/−^ mice produced equivalent chemokine concentrations, TNF^−/−^ mice synthesised significantly higher levels (*p* < 0.01) of MIP-1α, MCP-1 and RANTES at 3 weeks post-infection (Fig. 6d–f). The positive correlation between higher chemokine concentrations and enhanced cellular inflammation in TNF^−/−^ mice suggests an unregulated TNF-dependent chemokine response driving uncontrolled cell recruitment in response to *M. tuberculosis* infection. Thus, while immune cell recruitment and proliferation were TNF dependent, neuron-derived TNF was redundant for this function.

### TNF deficiency renders cerebral innate immune cells functionally defective

**Fig. 7:**
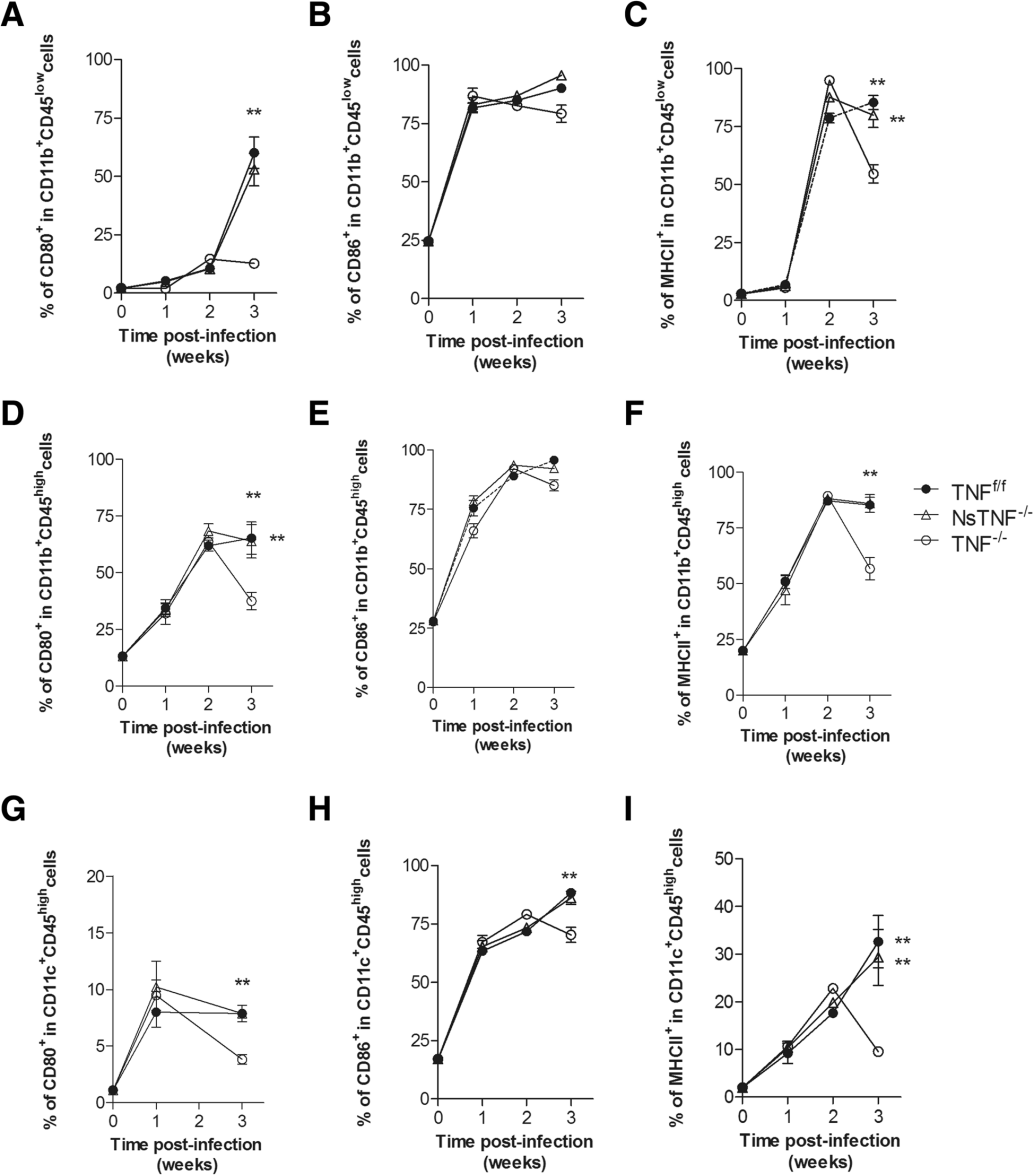
Expression of CD80, CD86 and MHCII in microglia, macrophages and dendritic cells is TNF dependent. TNF^f/f^ (*black circle*), NsTNF^−/−^ (*triangle*) and TNF^−/−^ (*clear circle*) mice were infected with *M. tuberculosis* H37Rv at a dose of 1 × 10^3^–1 × 10^4^ CFUs/brain by intracerebral inoculation. Single-cell suspensions of brains at 0, 1, 2 and 3 weeks post-infection were generated and microglia (**a**–**c**), macrophages (**d**–**f**) and dendritic cells (**g**–**i**) analysed for expression of CD80 (**a**, **d**, **f**), CD86 (**b**, **e**, **h**) and MHC II (**c**, **f**, **i**). Data (mean ± SEM of the CFUs of 5–6 mice per time point) are representative of repeat experiments. **p ≤ 0.01

The increased presence of innate immune cells in the absence of TNF supports the potential for improved early control of cerebral *M. tuberculosis*, yet TNF^−/−^ mice display a highly susceptible phenotype. We postulated that early susceptibility may be associated with defective function of innate immune cells during initial infection. We therefore investigated the capacity of microglia, macrophages and dendritic cells to present antigen by analysing expression of MHCII, CD80 and CD86 (Fig. 7). We observed comparable expression of CD80, CD86 and MHCII in TNF^f/f^ and NsTNF^−/−^ mice at each of the time points investigated, indicating that neuron-derived TNF does not regulate innate immune cell activation. In contrast, although TNF^−/−^ mice presented with equivalent levels of immune-activated cells during the first 2 weeks of infection compared to TNF^f/f^ and NsTNF^−/−^ mice, they fail to sustain the required level of activation and significantly lower (*p* < 0.05) levels of activated innate immune cells were measured at 3 weeks post-infection.

**Fig. 8:**
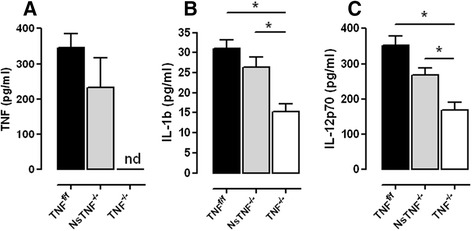
TNF regulates synthesis of IL-1β and IL-12p70. TNF^f/f^ (*black*), NsTNF^−/−^ (*grey*) and TNF^−/−^ (*clear*) mice were infected with *M. tuberculosis* H37Rv infection at a dose of 1 × 10^3^–1 × 10^4^ CFUs/brain by intracerebral inoculation. **a** TNF, **b** IL-1β and **c** IL-12p70 cerebral concentrations were measured by ELISA at 3 weeks post-infection. Data (mean ± SEM of the CFUs of 5–6 mice per time point) are representative of repeat experiments. *p ≤ 0.05

To further investigate innate immune cell functionality, we measured levels of TNF, IL-1β and IL-12p70 in the brain homogenates of TNF^f/f^, NsTNF^−/−^ and TNF^−/−^ mice after 3 weeks of intracranial infection (Fig. 8). The induction of TNF in TNF^f/f^ mice confirmed its requirement as part of initial protective immune responses, while equivalent expression in NsTNF^−/−^ mice indicated neurons redundant as a TNF source (Fig. 8a). As expected, no TNF production was detected in TNF^−/−^ mice (Fig. 8a). Similar levels of IL-1β (Fig. 8b) and IL-12p70 (Fig. 8c) production were observed in TNF^f/f^ and NsTNF^−/−^ infected brains. In contrast, the concentration of cerebral IL-1β and IL-12p70 in infected TNF^−/−^ mice was significantly (*p* < 0.05) lower than in TNF^f/f^ and NsTNF^−/−^ mice. Therefore, the data indicates that TNF is required to regulate cytokine synthesised by innate immune cells but that neuron-derived TNF is not important.

## Discussion

TNF concentration forms a pivotal point of balance that determines outcome of disease during *M. tuberculosis* infection. Susceptibility is promoted either under conditions where excess TNF prevails or low TNF concentrations are present [15, 16, 23, 24]. Under such polar conditions, either macrophage/cell effector function is impaired which leads to intracellular mycobacterial replication and eventual cell lysis or excessive TNF levels promote macrophage necrosis, in so doing enrich conditions for bacilli growth. Diverse cell types can act as contributory sources of TNF (during pathogenic challenge) and may therefore be differential contributors to the TNF environment required for optimum mycobacterial control. It is known that microglia, as resident immune cells of the CNS, express TNF and that recruited dendritic cells and macrophages will similarly induce TNF during pathogen challenge [40, 52, 53]. Neurons, as the single most abundant cell type within the CNS, are capable of regulating immune responses during CNS insult [54, 55]. We have reported *M. tuberculosis* targeting of neurons and its capability to generate reactive immune responses [42]; however, its role in *M. tuberculosis* control remains largely untested. In the presence of pathogens, neurons induce specific cytokine signatures, TNF comprising a key component of many such signature responses. The synthesis of TNF by neurons was observed after systemic immune challenge [56]. Upon viral infection, both in vitro and clinical studies have shown TNF expression in neurons [57–60]. In other neurological diseases, upregulation of neuronal TNF expression has provided an indication to the severity of disease progression and inflammatory neuropathology [43, 61–63]. In view of these findings, we postulated that neuron-derived TNF may have a contributory role to regulate CNS immunity during *M. tuberculosis* infection. We therefore investigated the overall contribution of TNF and in particular the involvement of neuron-derived TNF in host-mediated immunity directed against *M. tuberculosis* during central nervous system infection. Several converging lines of study/evidence have indicated a neuroprotective role for TNF and its induction demonstrated to be a key determinant of disease outcome during cerebral infection [64]. In this study, we initially demonstrated that global TNF is critical to control *M. tuberculosis* dissemination to the CNS and regulates immune-mediated brain pathology during infection. Moreover, we demonstrate for the first time that TNF is an absolute requirement to mediate protective immunity against CNS-TB; however, neuron-derived TNF is considered redundant. Thus, host susceptibility to CNS-TB in the absence of TNF corroborates its significance in neuroprotective immunity. Although the findings in this study appear contrasting to those of Tsenova et al., where TNF was reported to promote mycobacterial onset of disease and pathogenesis [16], it is not unexpected and may rather reflect opposite ends of the TNF functionality spectrum as previously reported [23, 24]. TNF concentration in dynamic homeostasis is absolutely essential for effective control of *M. tuberculosis* infection, a shift in balance that favours either excessive or reduced levels being detrimental to the host that promotes conditions for mycobacterial persistence.

Mycobacterial infection of the CNS generates a robust immune response characterised by both pro-inflammatory and anti-inflammatory immune cellular and cytokine profiles [65]. While microglia were reported to be the primary source of TNF during CNS infection [65], we and others have shown that different cell types have hierarchical importance as sources of TNF in determining the outcome of tuberculosis disease [37]. TNF derived from T cells critically defines resolution of pulmonary infection, while TNF from macrophages has a transient role to control *M. tuberculosis* replication [37]. Our findings however ascribe neurons as a redundant cellular source of TNF to control *M. tuberculosis* infection or regulate immune pathology and suggest a far greater role for other resident or recruited cells to produce TNF. Interestingly, others have reported that TNF produced by neurons is redundant for mediating inflammation but that membrane TNF expressed on astrocytes has a functional role [66].

Nonetheless, the overall requirement of TNF for control of both mycobacterial replication and immune-mediated pathology was evident and the rapid rate of host susceptibility suggested immune deficiency during early infection. Brain pathology, particularly at end-stage disease, was characterised by increased inflammation of dendritic cells and macrophages, and microglial proliferation which was underpinned by an enhanced pro-inflammatory chemokine environment in the absence of TNF. These observations align with earlier reports of a regulatory role for TNF in inflammation during *M. tuberculosis* infection [67]. In earlier studies, we reported on a deregulated overall cytokine and chemokine response in the absence of TNF during pulmonary tuberculosis [37]. We show here a neutrophil-dominant pathology in TNF^−/−^ mice also associates with a similar unregulated increase in chemokine synthesis during CNS-TB. Moreover, a lethal neutrophil-driven inflammatory response associated with, amongst others, MIP-1α in the absence of microRNA 223 in pulmonary tuberculosis was reported [68]. Taken together, the data suggest that perturbation of controlled host immune regulation which promotes a dominant neutrophilic response leads to increase susceptibility with fatal consequences. Selective inhibition of neutrophil recruitment under conditions of immune suppression during tuberculosis should therefore be considered.

Microglia, macrophages and dendritic cells as the main facilitators of early innate immune protection are activated in a TNF-dependent manner [69, 70]. We therefore postulated that early host susceptibility of TNF^−/−^ mice was the result of defective activation of innate antigen-presenting immune cells during early infection. Activation of macrophages, dendritic cells and microglia within the first 2 weeks of infection appeared to be independent of TNF with equivalent levels of activated cells evident in TNF^f/f^, NsTNF^−/−^ and TNF^−/−^ mice; however, the ability to subsequently sustain the required measures of activated cells was TNF dependent. These cells which are critical effectors of early innate immunity were rendered functionally defective in the absence of TNF. Initial cytokine signature responses by innate immune cells include synthesis of IL-12 and IL-1 [71, 72], both of which are critical to control tuberculosis infection [14, 73–75]. Mice deficient for either cytokine develop rapid onset of disease and succumb to infection, while patients with defective IL-12 signalling display susceptibility to tuberculosis infection. Maturation of antigen-presenting cells is characterised by the expression of, amongst others, MHCII and CD80 [76] during *M. tuberculosis* infection and is mediated largely by the presence of IL-1 and TNF [77, 78]. Moreover, IL-12 synthesis by mature activated dendritic cells is dependent on TNF-mediated signalling [78] and is a key cytokine required to restrict bacilli replication. Here, we observed that maturation of microglia, dendritic cells and macrophages initially occurs independent of TNF; however, sustained cell activation was TNF dependent. The deficiency in IL-12 and IL-1β synthesis in the CNS when TNF is absent argues strongly for functional failure of innate immune cells and inability to effectively participate in control of *M. tuberculosis*. Moreover, the reduction of MHCII- and CD80-expressing cells indicated reduced capacity to initiate *M. tuberculosis*-specific Th1 protective immune responses required for control of intracellular bacilli, and hence predisposed the host to a susceptible phenotype.

## Conclusion

In conclusion, the findings in this study corroborate earlier reports of the global importance of TNF in immune-mediated responses against tuberculosis and for the first time demonstrate its importance to control *M. tuberculosis* replication within the CNS. We further showed that neuron-derived TNF is not required for this process.

## Abbreviations

BBB: Blood–brain barrier
BCG: Bacillus Calmette–Guérin
CCL: Chemokine C–C motif ligand
CD: Cluster of differentiation
CFU: Colony-forming unit
CNS: Central nervous system
CNS-TB: Tuberculosis of the central nervous system
CSF: Cerebrospinal fluid
GFP: Green fluorescent protein
H&E: Haematoxylin and eosin
IL: Interleukin
LPS: Lipopolysaccharide
MAP: Microtubule-associated protein
MCP: Monocyte chemotactic protein
MHC: Major histocompatibility complex
MIP: Macrophage inflammatory protein
*M. tuberculosis*: *Mycobacterium tuberculosis*
NsTNF^−/−^: Neuron-specific TNF-deficient
TB: Tuberculosis
TNF: Tumour necrosis factor
TNF^−/−^: Tumour necrosis factor-deficient
TNF^f/f^: Tumour necrosis factor-floxed
